# Long-term post-trial extension of osilodrostat therapy in selected Canadian patients with Cushing disease from the LINC 3 and LINC 4 programs

**DOI:** 10.3389/fendo.2026.1902818

**Published:** 2026-10-12

**Authors:** Luciana Martel-Duguech, Ghislaine Houde, Constance Chik, Syed Ali Imran, André Lacroix

**Affiliations:** 1 Division of Endocrinology, Department of Medicine, Centre hospitalier de l’Université de Montréal, Montreal, QC, Canada; 2 Division of Endocrinology, Department of Medicine, University of Sherbrooke, Sherbrooke, QC, Canada; 3 Division of Endocrinology and Metabolism, Department of Medicine, University of Alberta, Edmonton, AB, Canada; 4 Division of Endocrinology and Metabolism, Dalhousie University, Halifax, NS, Canada

**Keywords:** adrenal insufficiency, Cushing disease (CD), LINC 3, LINC 4, long-term follow-up, osilodrostat

## Abstract

**Background:**

Long-term outcomes of selected patients who continue osilodrostat for Cushing disease after clinical-trial participation remain incompletely described.

**Methods:**

We retrospectively analyzed a selected multicenter post-trial cohort of Canadian patients with Cushing disease previously enrolled in LINC 3 (NCT02180217) or LINC 4 (NCT02697734) who continued osilodrostat in the global roll-over study and, when applicable, in a Canadian investigator-initiated extension phase (ISS-CA-2023; NCT06131580). The dataset included 11 Canadian osilodrostat-exposed LINC participants who entered post-trial roll-over follow-up; 7 subsequently entered the Canadian investigator-initiated extension, whereas 4 discontinued during earlier follow-up for defined reasons. For longitudinal efficacy analyses, baseline and time zero were defined as first osilodrostat exposure in the parent LINC study; week 14 refers to week 14 after osilodrostat initiation. The main outcomes were time to first mean urinary free cortisol (mUFC) control, durability of control, long-term dose evolution, pituitary tumor surveillance, and safety.

**Results:**

Eleven selected Canadian patients received continued osilodrostat, and all achieved first biochemical control after a median of 5 weeks (range: 2–12). Median mUFC decreased from 2.65 × ULN at parent-study baseline to 0.56 × ULN at week 14; these descriptive values were not used for inferential testing. Seven patients remained on therapy long term; at the last follow-up, six of seven had controlled mUFC after a median exposure of 98 months (range: 83 to 121). Maintenance doses varied widely, with a median last-visit dose of 6 mg/day (range: 0.125 to 40), and several patients required progressive down-titration over time. Hypocortisolism/adrenal-insufficiency events were common and generally managed with dose interruption or reduction and temporary glucocorticoid replacement. Clinically significant pituitary tumor enlargement leading to discontinuation and surgery occurred in two patients.

**Conclusion:**

In this selected Canadian post-trial continuation cohort, osilodrostat was associated with rapid initial biochemical control and durable long-term control in many patients for up to 10 years. The findings support the feasibility of long-term individualized osilodrostat management in selected patients continuing therapy after LINC 3/LINC 4 participation, with ongoing need for dose adaptation and surveillance for adrenal insufficiency and pituitary tumor behavior.

## Introduction

Cushing disease (CD) is a rare but deleterious endocrine disorder caused by chronic hypercortisolism secondary to an adrenocorticotropic hormone (ACTH)-secreting pituitary corticotroph adenoma, leading to excess morbidity and mortality (1–6). Transsphenoidal selective removal of the corticotroph adenoma remains first-line treatment; however, persistent or recurrent disease after surgery is frequent, and second-line therapy, including long-term medical treatment, is required in approximately 30%–40% of patients (3, 7, 8). Osilodrostat, an oral 11β-hydroxylase inhibitor, normalizes 24-h urinary free cortisol (UFC) and improves clinical outcomes in patients with CD in the pivotal phase III LINC 3 (NCT02180217) and LINC 4 (NCT02697734) studies (7, 9). LINC 3 was a 48-week, phase III, multicenter study with a 24-week open-label titration/treatment period followed by an 8-week randomized-withdrawal phase and subsequent open-label treatment to week 48. Osilodrostat was superior to placebo at maintaining mean urinary free cortisol (mUFC) ≤ ULN after randomized withdrawal and normalized mUFC in approximately two-thirds of enrolled patients by week 48 (9). In the LINC 3 extension, patients with investigator-assessed benefit at week 48 could continue open-label osilodrostat until all had received at least 72 weeks of treatment or discontinued; median exposure from core-study baseline was 130 weeks, and 81% of extension entrants had mUFC ≤ ULN at week 72 (10). LINC 4 was a 48-week phase III study with an upfront 12-week randomized, placebo-controlled period followed by 36 weeks of open-label osilodrostat and an optional extension initially intended to week 96; at the extension end-of-trial visit, 72.4% of patients had normal mUFC and reductions in serum cortisol and late-night salivary cortisol were maintained (11).

Longer-term evidence now extends beyond the pivotal trials and their extension phases. In a Polish single-center cohort of six patients with persistent or recurrent CD who initially participated in LINC 4, biochemical control referred to sustained normalization of median UFC/complete disease control through 156 weeks, with all six patients meeting the LINC 4 key secondary endpoint at week 36 and none excluded because of lack of effectiveness (13). In the US ILLUSTRATE real-world chart-review study of 42 patients with endogenous Cushing syndrome, biochemical improvement referred to reductions in cortisol measurements during routine care: UFC decreased below the ULN in 14 of 20 patients (70.0%) with pretreatment UFC above the ULN, and in the CD subgroup, median UFC decreased from 3.03 × ULN to 0.71 × ULN at the last assessment, with no unexpected safety signals (14). In a Spanish multicenter cohort of 37 patients with CD, response was defined as UFC normalization (complete response) or UFC reduction >50% without normalization (partial response); 33 patients (89.2%) had complete response and 4 had partial response (12). Nevertheless, continuous post-trial follow-up approaching a decade, with local biochemical monitoring and serial pituitary imaging, remains sparsely described.

## Methods

### Study design and participants

We conducted a retrospective multicenter cohort study of Canadian participants with CD who were exposed to osilodrostat in LINC 3 (*n* = 8) or LINC 4 (*n* = 3) and had post-parent-study follow-up in the global roll-over study (NCT03606408) and, when eligible, in a Canadian investigator-initiated extension (NCT06131580). The source dataset comprised 11 Canadian osilodrostat-exposed LINC participants who entered the post-trial continuation pathway. Of these, seven patients were still receiving osilodrostat when the global roll-over program closed and entered the Canadian investigator-initiated extension; the remaining four had discontinued during earlier post-trial follow-up after initial continuation, for the reasons described below. No additional Canadian osilodrostat-exposed LINC participants were available to the investigators for this analysis. The analytic cohort is therefore a selected post-trial continuation cohort enriched for patients with investigator-assessed benefit and/or tolerability sufficient to continue osilodrostat after the parent studies. The global roll-over study closed in December 2023 after commercial availability of osilodrostat in the United States and Europe. Because commercial access in Canada remained delayed during the study period, the seven Canadian patients with ongoing clinical benefit were offered continued treatment in a national investigator-initiated extension across four centers (Montreal, Sherbrooke, Edmonton, and Halifax). Follow-up was censored at the February 2025 data cutoff.

### Parent LINC protocols and time definitions

LINC 3 comprised 24 weeks of open-label osilodrostat titration/treatment, an 8-week randomized-withdrawal phase for eligible responders, and open-label treatment to week 48, followed by an optional extension for patients with clinical benefit. LINC 4 comprised an initial 12-week randomized double-blind placebo-controlled period, followed by open-label osilodrostat to week 48 and an optional extension initially intended to week 96. For patients continuing osilodrostat, there was no protocol-mandated washout between the parent LINC study, the global roll-over phase, and the Canadian extension; the only washout periods relevant to this cohort were the pre-LINC washouts from prior medical therapies required by the parent protocols. In this manuscript, baseline and time zero refer to the first exposure to osilodrostat in the original LINC study, unless explicitly stated otherwise. Week 14 and time to first mUFC control are therefore measured from osilodrostat initiation in the parent LINC study. Operational baseline visits for a given extension phase used the end-of-trial visit if within 1 month, or were repeated otherwise, but those extension-baseline visits were not used as the primary baseline for longitudinal efficacy analyses.

Patient flow was as follows: Canadian LINC 3/LINC 4 participants exposed to osilodrostat and entering global roll-over follow-up, *n* = 11; entered the Canadian investigator-initiated extension after global roll-over closure, *n* = 7; and discontinued before entry into the Canadian extension or before the February 2025 cutoff, *n* = 4 [2 for pituitary tumor progression and 2 for adverse events (AEs)]. Thus, the longest treatment-duration analyses reflect the seven patients who continued in the Canadian extension, whereas the discontinuation analyses describe the four patients withdrawn during earlier follow-up for known reasons.

### Treatment

Osilodrostat was continued per investigator judgment, with a maximum dose of 30 mg twice daily. Dose increases, reductions, or temporary interruptions followed protocol guidance. Visits occurred every 12 weeks (±2), with additional visits as clinically indicated. Osilodrostat was generally managed with an individualized titration approach. A block-and-replace strategy was not part of the original LINC 3 or LINC 4 titration protocols and was used later in follow-up for one patient (Case 9) according to investigator judgment to manage recurrent hypocortisolism while maintaining biochemical control.

### Biochemical assays and data harmonization

Biochemical monitoring combined central UFC data from the parent LINC and global roll-over phases with local measurements obtained during the Canadian investigator-initiated extension. During the parent LINC and global roll-over phases, UFC was measured by central-laboratory liquid chromatography–tandem mass spectrometry (LC-MS/MS) according to study protocols. The transition from central to local UFC assessment occurred with entry into the Canadian investigator-initiated extension in 2023. During the Canadian extension, UFC was measured by LC-MS/MS in all participating centers; CHUM and Sherbrooke used the same LC-MS/MS assay, and Edmonton and Halifax confirmed LC-MS/MS use for extension measurements. UFC was based on two 24-h collections per visit, averaged when both were available, and summarized as fold of the site-specific ULN to account for different reference intervals across central and local laboratories. Because UFC was measured by LC-MS/MS throughout, UFC-based endpoints are not subject to the specific immunoassay cross-reactivity issue from steroid precursors that may accumulate during CYP11B1 inhibition. Morning serum cortisol and ACTH were measured in accredited laboratories using local methods and reference ranges and were interpreted together with symptoms, dose changes, and local clinical context.

### Outcomes

Protocol objectives were safety (frequency and severity of AEs/SAEs) and investigator-assessed clinical benefit. Prespecified exploratory outcomes for this analysis included proportion with mUFC control (≤ ULN) and time and dose to first control.

### Safety definitions and monitoring

AEs/SAEs were collected continuously. Notable QTc findings followed protocol thresholds [>60 ms increase from baseline or QT interval corrected by Fridericia formula (QTcF) >500 ms confirmed], with confirmed QTcF >500 ms requiring discontinuation. Laboratory abnormalities were graded using CTCAE v5.0. Hypocortisolism-related events were summarized by clinical severity and management requirement. Events requiring temporary glucocorticoid replacement and/or osilodrostat dose interruption/reduction are distinguished from severe adrenal crisis, hospitalization for adrenal insufficiency, permanent discontinuation for hypocortisolism, or significant QT prolongation. Because central and local laboratory reference intervals differed during long-term follow-up, UFC was interpreted as fold-ULN and serum cortisol/ACTH were interpreted using local reference ranges and clinical context.

### Statistical analysis

Given the small sample size and selected continuation-cohort design, all analyses were descriptive. Continuous variables are presented as median (range) and categorical variables are presented as *n* (%). No *p*-values or formal between-group statistical comparisons were used to support conclusions; subgroup findings were interpreted descriptively and hypothesis-generating.

### Ethics

The ISS and all participating sites had local REB/IRB approvals; all patients provided written informed consent for continued treatment and data use in accordance with the protocol.

## Results

### Study population

Detailed individual and overall patient demographics and clinical characteristics at the parent LINC baseline (time zero) are summarized in **Table 1**. The study population reflects patients who entered continuation follow-up after trial participation and is therefore enriched for benefit/tolerability. Most patients (9/11) had previously undergone transsphenoidal surgery (TSS). Of the other two patients, one was treatment-naive and not found eligible for surgery because MRI showed bilateral carotid aneurysms in a partially empty sella, no visible adenoma, and mild hypercortisolism (Case 1); the second patient had only been treated with cabergoline before study inclusion (Case 10).

**Table 1:** Baseline characteristics stratified by osilodrostat status at the last follow-up.

Characteristic	Overall (*n* = 11)	Ongoing therapy (*n* = 7)	Discontinued therapy (*n* = 4)
Age, years, median (range)	41 (24–67)	41 (27–67)	42 (24–50)
Female sex, *n* (%)	9	5	4
Ethnicity, *n* (%) (Caucasian/Black/Asian/Other)	10/1/0/0	6/1/0/0	4/0/0/0
BMI, kg/m², median (range)	33 (21.3–45.9)	27 (21–44)	39.5 (21–44)
Diagnosis-to-osilodrostat interval, months, median (range)	48 (11–180)	54 (11–180)	39 (17–112)
Baseline mUFC, ×ULN, median (range)	2.65 (1.49–6.7)	2.6 (1.8–6.7)	2.5 (2.3–2.6)
Baseline mUFC severity, *n* (%): mild (<2 ×ULN)/moderate (2–5 ×ULN)/severe (>5×ULN)	2/7/2	2/3/2	0/4/0
Tumor size at diagnosis, *n* (%): macroadenoma (≥10 mm)/microadenoma or non-visible lesion (<10 mm)	3/8	3/4	0/4
Prior transsphenoidal surgery, *n* (%)	9	6	3
Prior pituitary radiotherapy, *n* (%)	1	1	0
Any prior medical therapy for hypercortisolism, *n* (%)	8	4	4

Baseline demographic, clinical, and biochemical characteristics of the overall cohort (*n* = 11), stratified by treatment status at the last follow-up (ongoing therapy, *n* = 7; discontinued therapy, *n* = 4). Continuous variables are presented as median (range), and categorical variables are presented as number (percentage). No formal statistical comparisons were performed because of the small sample size, *post-hoc* subgroup definition, and selected continuation-cohort design. Analyses were performed using available case data; BMI was missing for three patients and baseline mUFC was missing for one patient. Baseline refers to first osilodrostat exposure in the parent LINC study. BMI, body mass index; mUFC, mean urinary free cortisol; ULN, upper limit of normal; ACTH, adrenocorticotropic hormone.

### Osilodrostat dose and exposure, time to mUFC control, and mean urinary free cortisol levels over time

#### Osilodrostat dose and exposure in the 11 patients

Median osilodrostat exposure for the overall cohort was 84 months (range: 0.88–121 months) from first osilodrostat exposure in LINC 3/LINC 4, and the median average daily dose at week 14 after osilodrostat initiation was 6 mg/day (range: 2–25). Early dose changes were common, with the first adjustment at a median of 4 weeks (range: 2–24). Of the 11 patients, 5 had at least one interruption (4 within the first 12 weeks). Eight patients (73%) reached their individual maximum dose within 12 weeks; thereafter, 7/8 (87.5%) required dose reductions, most often within weeks 0–12 (5/8, 62.5), then weeks 12–24 (1/7, 14.3), weeks 24–36 (1/7, 14.3), and after week 72 (1/7, 14.3). Dose reductions were mainly triggered by hypocortisolism-related AEs (5/7), less often by severe edema (1/7) or significant lower-limb pain (1/7); one patient maintained the maximum dose to end of treatment. Of the 11 patients, 10 were managed primarily with titration. One patient (Case 9) later received a block-and-replace strategy during long-term follow-up; this was an investigator-adopted management strategy after parent LINC participation, not a routine LINC 3/4 protocol strategy.

#### Mean urinary free cortisol levels over time and time to mUFC control

All 11 patients achieved first biochemical control, defined as first mUFC normalization ≤ ULN from parent-study baseline, at a median of 5 weeks (range: 2–12): median week 6 for baseline mUFC <2 × ULN, week 4 for 2–5 × ULN, and week 6 for >5 × ULN. Median mUFC decreased from 2.65 × ULN at baseline (*n* = 11) to 0.56 × ULN at week 14 after osilodrostat initiation (*n* = 10), with 80% (8/10) controlled. The dose achieving first mUFC control varied, most commonly 4 mg/day (45%); median doses were 5.5 mg/day (range: 1–10) for baseline mUFC <2 × ULN, 7 mg/day (range: 4–20) for 2–5 × ULN, and 12 mg/day (range: 4–20) for >5 × ULN. These dose-stratum comparisons are descriptive only.

First, mUFC control and maximum early dose should be interpreted as different metrics. First control captured the earliest normalized mUFC, whereas dose titration during the first 12 weeks followed serial UFC values, clinical status, and tolerability. Some patients required further titration after an initial normalized collection because control had not yet been sustained or because subsequent UFC values and symptoms required adjustment. This explains why 8/11 patients reached their individual maximum dose within 12 weeks despite median first control at 5 weeks; seven of those eight subsequently required dose reductions.

### Patients who discontinued osilodrostat therapy (*n* = 4)

Four patients discontinued osilodrostat permanently during the study period (**Table 2**, **Figure 1**).

**Figure 1:**
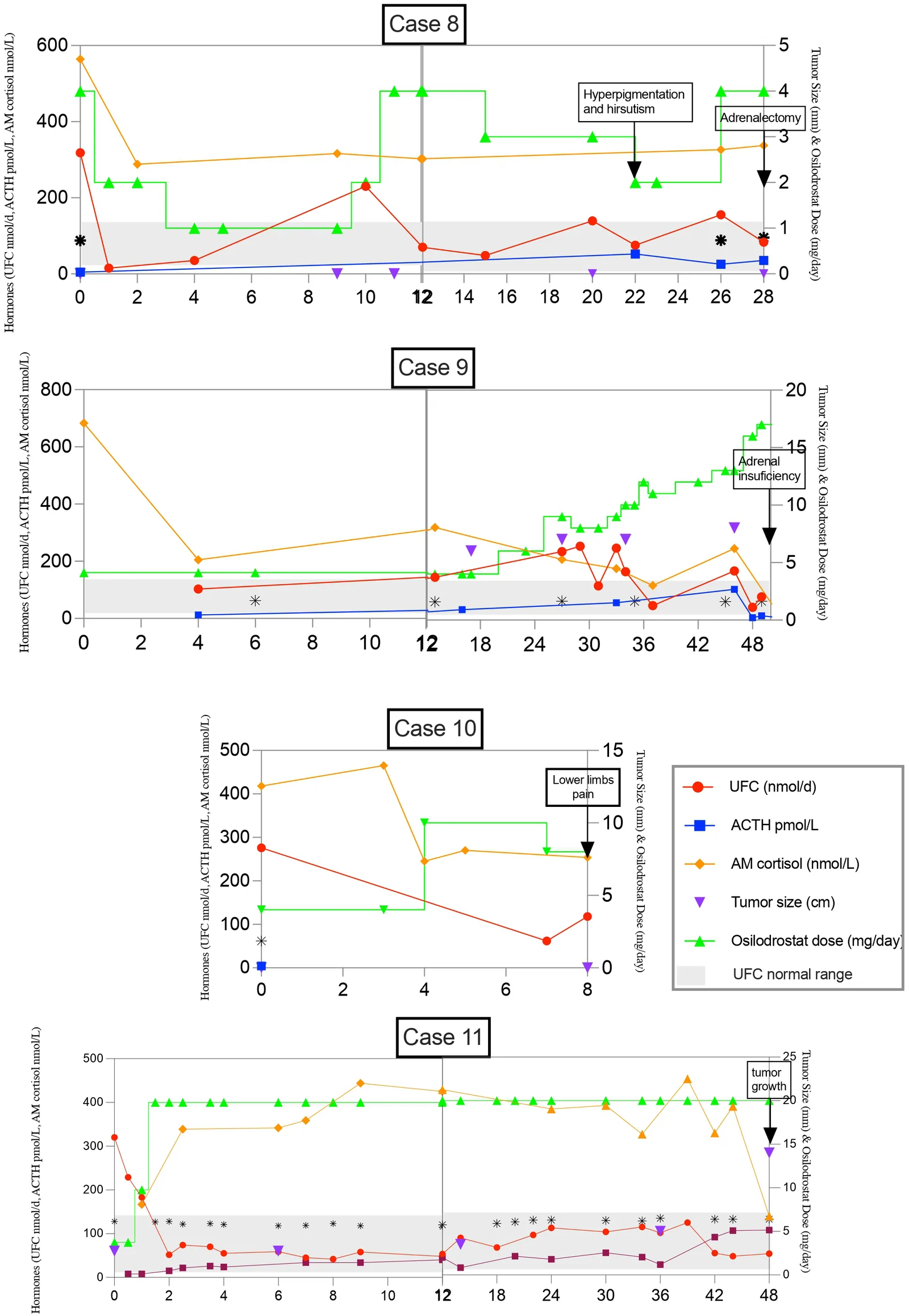
Osilodrostat discontinued (Cases 8 to 11). Individual longitudinal trajectories of biochemical measures, tumor size, and osilodrostat dose in patients who discontinued osilodrostat during follow-up. Each panel shows UFC, plasma ACTH, and morning serum cortisol on the left *y*-axis, and osilodrostat daily dose and tumor size on the right *y*-axis, plotted against time (months). The vertical line at 12 months (when present) indicates the transition from the early titration phase to long-term follow-up. The shaded band indicates the UFC reference range. Arrows indicate major adverse events or outcomes temporally associated with discontinuation (for example, hyperpigmentation and hirsutism, adrenal insufficiency, adrenalectomy, lower-limb pain, and tumor growth). Black asterisks indicate periods of glucocorticoid replacement; in Case 9, this corresponded to a later investigator-adopted block-and-replace approach during long-term follow-up, not a parent LINC protocol strategy.

#### Osilodrostat dose and exposure

Median osilodrostat exposure in this subgroup was 27 months (range: 5–51). The median average daily dose was 3.2 mg/day (range: 2.42–10.8), and the median dose administered for the longest duration was 11 mg/day (range: 1.5–20).

#### mUFC levels and time to mUFC control

Initial biochemical control occurred at a median of 5 weeks after osilodrostat initiation in the parent LINC study (range: 2–12). The proportion of patients with mUFC normalization (≤ ULN) was 75% (3/4) at week 14, declining to 67% (2/3) at week 72 and 34% (1/3) at week 96. Mean mUFC decreased from 2.5 × ULN at baseline (range: 2.3–2.6) to 0.48 × ULN at week 14 (range: 0.25–1.36), then increased to 1.01 × ULN at week 72 (range: 0.46–1.72) and 1.12 × ULN at week 96. The median dose at first UFC normalization was 7 mg/day (range: 4–20), with a maximum administered dose of 12.5 mg/day (range: 4–20). After week 14, the median dose remained at 6 mg/day, with ranges of 4–20 mg/day at week 14, 3–20 mg/day at week 72, and 1.5–20 mg/day at week 96. Marked interindividual variability was observed (CV 84.1% at week 14, 93.8% at week 72, and 105.2% at week 96). All four patients had at least one dose interruption, mainly within the first 12 weeks; the median number of dose adjustments was 8 per patient (range: 3–15), with median minimum and maximum doses of 3.5 mg/day (range: 1–4) and 8.5 mg/day (range: 3–15), respectively.

Reasons for discontinuation were AEs in two patients (hirsutism with hyperpigmentation and significant lower-limb pain) and clinically significant pituitary tumor progression in two patients, including one with progression despite normal UFC on osilodrostat. All patients underwent subsequent definitive management, including adrenalectomy, TSS, and radiotherapy when indicated, and all were controlled or in remission at the last follow-up. Individual discontinuation details and outcomes are summarized in **Table 2**.

**Table 2:** Patients who discontinued osilodrostat therapy.

Patient ID	Time of osilodrostat treatment (months)	Phase of discontinuation	Reason for discontinuation	Outcome/Follow-up
8	27	Rollover, CLCI699-2X01B, week 36	Hirsutism and hyperpigmentation	Bilateral adrenalectomy +Gamma knife to 3 mm pituitary lesion. Controlled
9	51	Rollover, CLCI699-2X01B, week 24	Tumor progression	TSS of recurring pituitary corticotroph adenoma. In remission
10	5	Initial phase CLCI 2301	Lower-limb pain	Medical treatment (cabergoline) and TSS 4 years after. In remission
11	48	Extension phase	Tumor progression despite normal cortisol levels on Osilodrostat	TSS. In remission (Fontaine-Sylvestre et al., 2021)

Summary of individual patients who discontinued osilodrostat, including duration of treatment prior to discontinuation, specific reasons for stopping therapy, and subsequent interventions and outcomes. TSS, transsphenoidal surgery.

### Long-term osilodrostat exposure in patients who continued therapy (*n* = 7)

Seven patients continued osilodrostat therapy through the last follow-up (**Table 3**, **Figure 2**).

**Figure 2:**
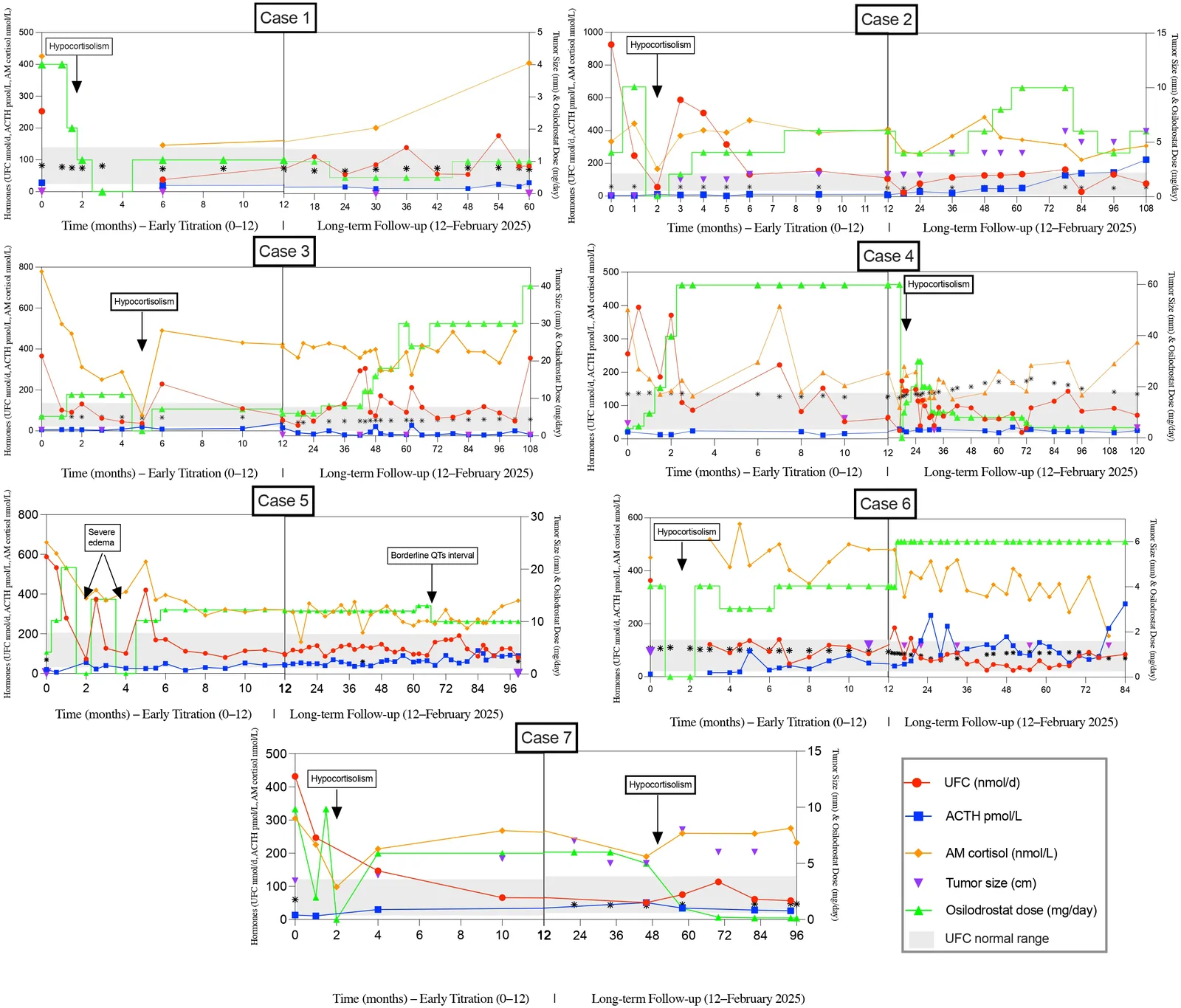
Ongoing osilodrostat treatment (Cases 1 to 7). Individual longitudinal trajectories of biochemical control, tumor size, and osilodrostat dose in patients remaining on osilodrostat at the last follow-up (through February 2025). Each panel shows urinary free cortisol (UFC), plasma ACTH, and morning serum cortisol on the left *y*-axis, and osilodrostat daily dose and tumor size on the right *y*-axis, plotted against time (months) from first osilodrostat exposure in the parent LINC study. The vertical line at 12 months separates the early titration phase (0 to 12 months) from long-term follow-up (12 months to February 2025), when applicable. The shaded band indicates the UFC reference range. Arrows highlight key adverse events or clinically relevant milestones (for example, hypocortisolism, severe edema, and borderline QTc prolongation). Black asterisks indicate periods of glucocorticoid replacement used to prevent or treat hypocortisolism.

#### Osilodrostat dose and exposure

Median osilodrostat exposure was 98 months (range: 83–121), with a median average daily dose of 6 mg/day (range: 1.39–16.5); the median dose used for the longest duration was also 6 mg/day (range: 0.0–60.0). Median dose at first UFC normalization was 4 mg/day (range: 1–20), and the median maximum dose during follow-up was 15 mg/day (range: 4–60). Dose requirements remained highly individualized over time, with marked interindividual variability (CV 146.2% at week 14, 152.8% at week 72, 81.9% at week 96, and 144.1% at the last follow-up). Patients required a median of 8 dose adjustments (range: 3–15), with median minimum and maximum doses of 3.5 mg/day (range: 1–4) and 8.5 mg/day (range: 3–15); median dose reductions were 50% and median dose increases 100%. At the last follow-up, the median dose was 6 mg/day (range: 0.125–40), corresponding to a 20% median change from baseline.

#### Mean UFC levels over time and time to UFC control

Initial biochemical control (UFC ≤ ULN) was achieved at a median of 5 weeks (range: 2–10) after osilodrostat initiation. Median mUFC decreased from 2.65 × ULN (range: 1.8–6.7) at baseline to 0.69 × ULN (0.28–2.28) at week 14 and remained stable at week 72 (0.57 × ULN, 0.36–1.24), week 96 (0.55 × ULN, 0.43–1.05), and the last follow-up (0.62 × ULN, 0.18–3.08). Sustained UFC normalization was observed in 85.7% at weeks 14 and 72, 71.4% at week 96, and 85.7% at final assessment. Detailed biochemical responses, dosing, and tumor status are summarized in **Table 3**.

**Table 3:** Patients with ongoing osilodrostat therapy: long-term treatment duration, current dose, biochemical control, and tumor status.

Patient ID	Duration on osilodrostat (months)	Current daily dose (mg/day)	Last mUFC (nmol/24 h)	UFC lab reference range (nmol/24 h)	Last ACTH (pmol/L; 2.0 to 11.0)	Cortisol AM (140 to 550 nmol/L)	Last MRI status (tumor size/changes)
1	83	0.5	86	<120	43	405	Stable arachnoidocele and pituitary atrophy
2	102	9	80	<120	202	284	Stable 6 mm
3	104	25	33	<120	6	497	Non-visible
4	121	4	68	<120	22	288	Stable at 4 mm
5	98	10	83.5	<160	93.5	373	Stable at 4 mm
6	84	6	86	12–486	>278	156.2	Stable at 1.4 mm
7	96	0.125	56	<120	26.4	228	Stable/shrinkage/increase

Individualized data for patients continuing osilodrostat, including treatment duration, current daily osilodrostat dose, last urinary free cortisol (UFC), ACTH, and morning cortisol values along with respective laboratory reference ranges. All UFC values shown were measured by LC-MS/MS and interpreted using the corresponding site-specific reference interval; ACTH and morning cortisol were interpreted using local reference ranges. MRI outcomes indicate tumor size status at the last follow-up.

#### Tumor volume

Pituitary tumor surveillance was performed by local MRI review. Progression was defined pragmatically as a >20% increase in maximal tumor diameter compared with the prior or baseline measurement when serial dimensions were available; volume was recorded when available but was not available with sufficient consistency to support formal volumetric progression analyses. Radiological increases >20% were observed after 12 months (2 observations), 24 months (1), 48 months (1), and 56 months (1), with a median time to documented increase of 24 months (range: 3–54). Tumor volume decreased by ≥20% from baseline in one patient. In this small cohort, ACTH elevations were frequent during CYP11B1 inhibition and no consistent temporal relationship between ACTH rise and radiological tumor growth could be established. Two patients had clinically significant tumor enlargement that led to osilodrostat discontinuation and TSS; available details are summarized in **Table 4**. Tumor progression occurred despite biochemical control in at least one patient, supporting continued pituitary imaging independent of UFC status.

**Table 4:** Clinically significant pituitary tumor progression during long-term osilodrostat therapy.

Patient ID	Time/progression description	Biochemical/ACTH context	Management and outcome
9	51 months; recurrent pituitary corticotroph adenoma with clinically significant enlargement during roll-over follow-up; progression was assessed locally by MRI.	No consistent ACTH-UFC temporal relationship could be established from available data.	Osilodrostat discontinued; TSS performed for recurrent corticotroph adenoma; in remission at the last follow-up.
11	48 months; tumor growth during extension follow-up while cortisol remained controlled on osilodrostat.	Progression occurred despite normal cortisol levels; ACTH increased during CYP11B1 inhibition, but causality cannot be inferred.	Osilodrostat discontinued; TSS performed; in remission after surgery (Fontaine-Sylvestre et al., 2021).

Patient-level summary of tumor progression events that led to osilodrostat discontinuation or pituitary surgery. MRI assessments were reviewed locally. ACTH, adrenocorticotropic hormone; mUFC, mean urinary free cortisol; TSS, transsphenoidal surgery; UFC, urinary free cortisol; ULN, upper limit of normal.

### Overview of adverse events (related or unrelated to osilodrostat)

AEs, classified by clinical category and management consequence, are detailed in **Table 5**. The predominant safety issue was hypocortisolism/adrenal insufficiency. Five of 11 patients had investigator-coded adrenal insufficiency events requiring temporary glucocorticoid replacement and dose interruption or reduction; none experienced adrenal crisis, hospitalization for severe adrenal insufficiency, permanent discontinuation due to hypocortisolism, or significant QT prolongation. Non-serious events were generally infrequent and rarely required dose modification, with dose adjustment reported mainly for mild hypocortisolism symptoms, edema, and hirsutism or hyperpigmentation.

**Table 5:** Adverse events reported during osilodrostat therapy.

Severity and adverse event	Patients, *n* (%)	Dose adjustment, *n* (%)	Discontinuation, *n* (%)
Protocol-recorded serious or clinically significant events			
Adrenal insufficiency requiring temporary glucocorticoid replacement and/or dose interruption/reduction	5 (45.5%)	5 (45.5%)	0 (0%)
Severe adrenal crisis or hospitalization for hypocortisolism/adrenal insufficiency	0 (0%)	0 (0%)	0 (0%)
Pituitary tumor enlargement requiring second pituitary surgery	2 (18.2%)	0 (0%)	2 (18.2%)
Significant QT prolongation	0 (0%)	0 (0%)	0 (0%)
Non-serious or non-severe events			
Mild hypocortisolism symptoms not meeting severe-event criteria	10 (90.9%)	1 (9.1%)	0 (0%)
Headache	2 (18.2%)	0 (0%)	0 (0%)
Lower-limb pain	2 (18.2%)	0 (0%)	1 (9.1%)
Arthralgia	3 (27.3%)	0 (0%)	0 (0%)
Edema	2 (18.2%)	1 (9.1%)	0 (0%)
Nasopharyngitis	0 (0%)	0 (0%)	0 (0%)
Hypertension	3 (27.3%)	0 (0%)	0 (0%)
Hirsutism	2 (18.2%)	1 (9.1%)	1 (9.1%)
Hyperpigmentation	1 (9.1%)	1 (9.1%)	0 (0%)
Psychiatric symptoms (depression/anxiety/panic attack)	5 (45.5%)	0 (0%)	0 (0%)

Adverse events categorized by clinical severity and management. Hypocortisolism-related categories are not mutually exclusive; five patients had events coded as adrenal insufficiency/protocol-reportable because they required temporary glucocorticoid replacement and/or dose interruption or reduction, but no patient had adrenal crisis, hospitalization for severe adrenal insufficiency, permanent discontinuation due to hypocortisolism, or QTcF >500 ms. QTcF, Fridericia-corrected QT interval.

One patient (Case 3) recently experienced a temporary interruption of osilodrostat therapy due to an unplanned pregnancy, which occurred following removal of an intrauterine device without prior discussion with the treating team. Osilodrostat was promptly discontinued, and a request for access to metyrapone was submitted to Health Canada. The pregnancy was subsequently complicated by a ruptured ectopic gestation, resulting in termination. Osilodrostat therapy was resumed thereafter. Because of lack of biochemical control, the dose was recently increased from 20 to 25 mg twice daily; at the most recent visit, mUFC was normalized at the low end of the reference range without symptoms of adrenal insufficiency, and close follow-up is ongoing.

## Discussion

This selected multicenter post-trial continuation cohort extends the available evidence on osilodrostat in CD by describing the Canadian LINC 3/LINC 4 continuation experience, including seven patients who entered the Canadian investigator-initiated extension and were treated for 83 to 121 months, together with patient-level information on the four remaining Canadian participants who continued osilodrostat during earlier post-trial follow-up and discontinued for defined reasons. The main contribution of this series is not to re-demonstrate short-term efficacy, which has already been established in the LINC trials and their extensions, but to illustrate long-term management among selected patients who continued therapy after trial participation. In particular, the cohort highlights durable biochemical control in many patients, marked heterogeneity in maintenance dose requirements, progressive down-titration to very low doses in some individuals, and the continued need to monitor both hypocortisolism/adrenal insufficiency and pituitary tumor behavior. These findings complement the LINC extension studies and more recent peer-reviewed real-world cohorts (12–14) by providing detailed follow-up in a selected cohort of patients who remained eligible for continuation treatment after parent-study participation.

These findings should be interpreted alongside newer post-trial and real-world studies. In the Polish single-center cohort, six patients with persistent or recurrent CD remained under complete biochemical control throughout 156 weeks of osilodrostat treatment (13). In ILLUSTRATE, 42 patients with endogenous Cushing syndrome were treated in routine practice over a median treatment duration of 37 weeks (range: 0.7–78.1); UFC fell below the ULN in 14 of 20 patients with pretreatment UFC above the ULN, and no unexpected safety signals were observed (14). In a Spanish multicenter cohort, 37 patients with CD were treated for a median of 5 months (range: 1–93), and all were classified as responders, including 33 patients (89.2%) with complete UFC normalization and 4 with partial response (12). Compared with these reports, the main added value of the present series is substantially longer continuous follow-up in the seven patients who entered the Canadian investigator-initiated extension, allowing better characterization of late dose de-escalation and long-term surveillance issues.

All patients achieved initial biochemical control within a median of 5 weeks on a median osilodrostat dose of 4 mg/day, closely matching response times reported in the LINC studies (approximately 35–41 days) (7, 9). This endpoint represents first biochemical control rather than stable long-term control. During early titration, dosing was adjusted according to serial mUFC, symptoms, and tolerability, and several patients required further titration or subsequent reduction after an initial normalized UFC collection. Higher baseline mUFC was associated descriptively with higher doses required to achieve first control, consistent with prior LINC data and supporting severity-based dose individualization (7). Beyond this early phase, long-term dosing showed marked interindividual variability, with maintenance doses ranging from 0.125 to 40 mg/day, consistent with the wide dose distributions, early titration adjustments, and titration-related AEs reported in LINC 3 and LINC 4 extension and pooled analyses (10, 11, 16, 17). Additionally, pooled analysis of LINC 3 and LINC 4 showed rapid and sustained improvements in blood pressure and glycemic parameters in patients with baseline hypertension or diabetes, supporting the broader clinical relevance of sustained cortisol normalization during osilodrostat therapy (18).

This heterogeneity is consistent with the LINC studies and supports the modified Delphi guidance for flexible, patient-specific titration based on biochemical control, clinical status, and tolerability rather than fixed algorithms (19–22). More recent practical guidance also emphasizes individualized titration, close monitoring for adrenal insufficiency, and selective use of the block-and-replace strategy in carefully chosen patients rather than as a routine strategy (22–24). In addition, patients should receive education on symptoms of adrenal insufficiency, emergency steroid card use, and access to oral or injectable hydrocortisone for intercurrent illness or major stress (22, 24).

Two patients in our series maintained normal mUFC on very low intermittent doses (1 mg every 2 days, Case 1; and 1 mg every 8 days, Case 7), whereas others required up to 50–60 mg/day during titration. This observation is clinically interesting but remains hypothesis-generating and should not be interpreted as evidence of adrenal remodeling or a persistent structural adrenal effect. Although delayed recovery of cortisol secretion after osilodrostat withdrawal has been reported (20–26), and adrenal volume reduction has been described in a recent case series (15), our cohort did not include systematic adrenal imaging, longitudinal steroid precursor profiling, or formal adrenal reserve testing. Consequently, the data cannot establish whether very-low-dose requirements reflect osilodrostat exposure, baseline disease severity, intrinsic sensitivity to CYP11B1 inhibition, prior treatments, or other patient-specific factors. The practical implication is limited to individualized dose de-escalation, symptom-guided monitoring for adrenal insufficiency, and reassessment of adrenal reserve after treatment interruption or discontinuation.

Case 11 provides a single clinical observation of persistent glucocorticoid dependence after osilodrostat withdrawal, pituitary surgery, and radiotherapy, with preserved ACTH responsiveness to desmopressin but no serum cortisol rise. This finding is compatible with impaired adrenal cortisol output, but causality cannot be assigned to osilodrostat and no conclusion can be drawn regarding adrenal remodeling or a persistent structural adrenal effect.

Key limitations include the small, retrospective, uncontrolled design and strong selection by continuation into the post-trial roll-over pathway and, for the longest follow-up, into the Canadian investigator-initiated extension. The cohort was enriched for patients with perceived clinical benefit and/or tolerability, which limits inference about long-term persistence, effectiveness, or safety in broader CD populations. The source dataset available to the investigators did not permit comprehensive analysis of Canadian patients who did not enter post-trial continuation follow-up. UFC was measured by LC-MS/MS throughout central and local phases; therefore, the specific concern of UFC immunoassay cross-reactivity with osilodrostat-induced steroid precursors does not apply. However, central and local data were generated in different laboratory settings and interpreted using site-specific reference intervals, so fold-ULN harmonization cannot remove all between-laboratory variability. Morning serum cortisol and ACTH were measured using local methods and interpreted in clinical context. Local MRI review, missing data, and inconsistent volumetric measurements may introduce variability in tumor-change analyses. The adrenal suppression observations are hypothesis-generating only; systematic adrenal imaging, longitudinal steroid profiling, and formal adrenal reserve testing would be required before any inference regarding adrenal remodeling or persistent structural adrenal effects. Therefore, estimates of control, durability, dose evolution, safety, and tumor change should be considered exploratory.

In conclusion, this selected post-trial multicenter continuation cohort shows that osilodrostat can provide durable biochemical control for many years in selected patients with CD who continued treatment after LINC 3/LINC 4 participation. The longest follow-up derives from seven patients who entered the Canadian investigator-initiated extension, whereas the four discontinued cases provide complementary patient-level information on discontinuation, tumor progression, and AE management. The principal long-term management lessons are the marked variability in maintenance dose requirements, the frequent need for dose down-titration over time, and the importance of ongoing monitoring for hypocortisolism/adrenal insufficiency and pituitary tumor behavior.

## Data Availability

The raw data supporting the conclusions of this article will be made available by the authors, without undue reservation.

## References

[1] Fleseriu M Auchus R Bancos I Ben-Shlomo A Bertherat J Biermasz NR . Consensus on diagnosis and management of Cushing’s disease: a guideline update. Lancet Diabetes Endocrinol. (2021) 9:847–75. doi: 10.1016/S2213-8587(21)00235-7 34687601 PMC8743006

[2] Gadelha M Gatto F Wildemberg LE Fleseriu M . Cushing’s syndrome. Lancet. (2023) 402:2237–52. doi: 10.1016/S0140-6736(23)01961-X 37984386

[3] Nieman LK Castinetti F Newell-Price J Valassi E Drouin J Yutaka T . Cushing syndrome. Nat Rev Dis Primers. (2025) 11:1–25. doi: 10.1038/s41572-024-00588-w 39848955

[4] Braun LT Vogel F Reincke M . Long-term morbidity and mortality in patients with Cushing’s syndrome. J Neuroendocrinol. (2022) 34:e13113. doi: 10.1111/JNE.13113 35312199

[5] Limumpornpetch P Morgan AW Tiganescu A Baxter PD Nyaga VN Pujades-Rodriguez M . The effect of endogenous Cushing syndrome on all-cause and cause-specific mortality. J Clin Endocrinol Metab. (2022) 107:2377–88. doi: 10.1210/CLINEM/DGAC265 35486378 PMC9282270

[6] Webb SM Valassi E . Quality of life impairment after a diagnosis of Cushing’s syndrome. Pituitary. (2022) 25:768–71. doi: 10.1007/S11102-022-01245-9 35767164 PMC9587961

[7] Gadelha M Bex M Feelders RA Heaney AP Auchus RJ Gilis-Januszewska A . Randomized trial of osilodrostat for the treatment of Cushing disease. J Clin Endocrinol Metab. (2022) 107:E2882–95. doi: 10.1210/CLINEM/DGAC178 35325149 PMC9202723

[8] Hinojosa-Amaya JM Cuevas-Ramos D . The definition of remission and recurrence of Cushing’s disease. Best Pract Res Clin Endocrinol Metab. (2021) 35:101485. doi: 10.1016/J.BEEM.2021.101485 33472761

[9] Pivonello R Fleseriu M Newell-Price J Bertagna X Findling J Shimatsu A . Efficacy and safety of osilodrostat in patients with Cushing’s disease (LINC 3): a multicentre phase III study with a double-blind, randomised withdrawal phase. Lancet Diabetes Endocrinol. (2020) 8:748–61. doi: 10.1016/S2213-8587(20)30240-0 32730798

[10] Fleseriu M Newell-Price J Pivonello R Shimatsu A Auchus RJ Scaroni C . Long-term outcomes of osilodrostat in Cushing’s disease: LINC 3 study extension. Eur J Endocrinol. (2022) 187:531–41. doi: 10.1530/EJE-22-0317 35980235 PMC9513654

[11] Gadelha M Snyder PJ Witek P Bex M Belaya Z Turcu AF . Long-term efficacy and safety of osilodrostat in patients with Cushing’s disease: results from the LINC 4 study extension. Front Endocrinol. (2023) 14. doi: 10.3389/FENDO.2023.1236465 37680892 PMC10482037

[12] Araujo-Castro M García-Centeno R González L Hanzu FA Orois A Camara R . Real-world data on the efficacy and safety of osilodrostat in patients with Cushing’s disease in Spain. J Clin Med. (2025) 14:7575. doi: 10.3390/JCM14217575 41226972 PMC12608593

[13] Dzialach L Sobolewska J Respondek W Szamotulska K Witek P . Cushing’s disease: Long-term effectiveness and safety of osilodrostat in a Polish group of patients with persistent hypercortisolemia in the experience of a single center. Biomedicines. (2023) 11:3227. doi: 10.3390/BIOMEDICINES11123227 38137448 PMC10741245

[14] Fleseriu M Auchus RJ Huang W Spencer-Segal JL Yuen KCJ Dacus KC . Osilodrostat treatment of Cushing syndrome in real-world clinical practice: findings from the ILLUSTRATE study. J Endocr Soc. (2025) 9:bvaf046. doi: 10.1210/JENDSO/BVAF046 40226519 PMC11986586

[15] Varlamov EV Park BJ Fleseriu M . Osilodrostat-associated adrenal gland shrinkage: a case series of patients with ACTH-dependent Cushing’s syndrome. J Clin Endocrinol Metab. (2026) 111(4):1084–1090. doi: 10.1210/CLINEM/DGAF552 41054820

[16] Newell-Price J Fleseriu M Pivonello R Feelders RA Gadelha MR Lacroix A . Improved clinical outcomes during long-term osilodrostat treatment of Cushing disease with normalization of late-night salivary cortisol and urinary free cortisol. J Endocr Soc. (2024) 9:bvae201. doi: 10.1210/JENDSO/BVAE201 39610378 PMC11604051

[17] Fleseriu M Pivonello R Lacroix A Biller BMK Feelders R Gadelha M . Osilodrostat dose impact on efficacy/safety in Cushing’s disease: large, pooled analysis of LINC 2, 3, and 4. Eur J Endocrinol. (2025) 193:606–17. doi: 10.1093/EJENDO/LVAF207 41052284

[18] Samson SL Donegan D Geer EB Gordon MB Hamidi O Huang W . Management of osilodrostat therapy in patients with Cushing’s syndrome: a modified Delphi consensus panel. J Endocr Soc. (2025) 9:bvaf103. doi: 10.1210/JENDSO/BVAF103 40620482 PMC12227144

[19] Antonini S Brunetti A Zampetti B Boeris D Saladino A Cozzi RC . Osilodrostat in Cushing’s disease: the management of its efficacy and the pitfalls of post-surgical results. Endocrinology Diabetes Metab Case Rep. (2022) 2022:22-0311. doi: 10.1530/EDM-22-0311 36515363 PMC9716402

[20] Castinetti F Amodru V Brue T . Osilodrostat in Cushing’s disease: the risk of delayed adrenal insufficiency should be carefully monitored. Clin Endocrinol. (2023) 98:629–30. doi: 10.1111/CEN.14551 34219265

[21] Poirier J Bonnet-Serrano F Thomeret L Bouys L Bertherat J . Prolonged adrenocortical blockade following discontinuation of osilodrostat. Eur J Endocrinol. (2023) 188:K29–32. doi: 10.1093/EJENDO/LVAD060 37300549

[22] Ferriere A Salenave S Puerto M Young J Tabarin A . Prolonged adrenal insufficiency following discontinuation of osilodrostat treatment for intense hypercortisolism. Eur J Endocrinol. (2024) 190:L1–3. doi: 10.1093/EJENDO/LVAD167 38123490

[23] Dzialach L Respondek W Siejka A Witek P . Prolonged adrenal suppression after osilodrostat discontinuation in a patient with Cushing’s disease with eventual hypercortisolism relapse: case report and literature review. Front Med. (2025) 12. doi: 10.3389/FMED.2025.1629387 41030260 PMC12477154

[24] Kaniuka-Jakubowska S Kunc M Maksymowicz M Łapiński M Kowalczyk A Kłosowski P . A case of prolonged adrenal insufficiency following osilodrostat discontinuation with a review of the literature. Endocrine J. (2026) 73(1):87–92. doi: 10.1507/ENDOCRJ.EJ25-0150 41139506 PMC12819063

[25] Veloski C Sturgeon A Hallanger Johnson J . Prolonged adrenal insufficiency after failed cryoablation and osilodrostat for Cushing syndrome in nodular adrenal disease. JCEM Case Rep. (2025) 3:91. doi: 10.1210/JCEMCR/LUAF091 40303510 PMC12037973

[26] Tejani S Abramowitz J Tritos NA Hamidi O Mirfakhraee S . Prolonged adrenal insufficiency after osilodrostat exposure with eventual recovery of adrenal function. JCEM Case Rep. (2024) 2:luae088. doi: 10.1210/JCEMCR/LUAE088 38832004 PMC11146141

